# Biosorption of Hexavalent Chromium from Aqueous Medium with *Opuntia* Biomass

**DOI:** 10.1155/2014/670249

**Published:** 2014-04-03

**Authors:** José A. Fernández-López, José M. Angosto, María D. Avilés

**Affiliations:** Department of Chemical and Environmental Engineering, Technical University of Cartagena (UPCT), 52 Paseo Alfonso XIII, 30203 Cartagena, Spain

## Abstract

The biosorption of hexavalent chromium from aqueous solutions by *Opuntia* cladodes and ectodermis from cactus fruits was investigated. Both types of biomass are considered low-cost, natural, and ecofriendly biosorbents. Batch experiments were carried out to determine Cr(VI) biosorption capacity and the efficiency of the biosorption process under different pH, initial Cr(VI) concentration, and sorbent dosage. The biosorption of Cr(VI) by *Opuntia* biomass was highly pH dependent, favoring higher metal uptake at low pH. The higher biosorption capacity was exhibited at pH 2. The optimal conditions were obtained at a sorbent dosage of 1 g L^−1^ and initial metal concentration of 10 mg L^−1^. Biosorption kinetic data were properly fitted with the pseudo-second-order kinetic model. The rate constant, the initial biosorption rate, and the equilibrium biosorption capacity were determined. The experimental equilibrium data obtained were analyzed using two-parameter isotherm models (Langmuir, Freundlich, and Temkin). The Langmuir maximum monolayer biosorption capacity (*q*_max_) was 18.5 mg g^−1^ for cladodes and 16.4 mg g^−1^ for ectodermis. The results suggest that *Opuntia* biomass could be considered a promising low-cost biosorbent for the ecofriendly removal of Cr(VI) from aqueous systems.

## 1. Introduction

The increased industrial activities, indiscriminate use of organic and inorganic fertilizers and pesticides, and disposal of industrial effluents enhance the possibility of pollution and toxicity of heavy metals in environment. Due to their extended persistence in biological systems and tendency to bioaccumulate, the contamination of water by toxic heavy metals is a worldwide environmental hazard [1, 2]. Chromium, with its great economic importance in industrial use is one of the major metal pollutants and, in the last few decades, the amount of chromium in aquatic and terrestrial ecosystems has increased as a consequence of human activities. The discharge of effluents by a variety of industries such as leather tanning, textile dyeing, electroplating, pigment manufacturing, refineries, wood preservative treatment, and steel fabrication constitutes one of the major causes of water pollution by chromium compounds [3–6], gaining great significance to detoxify them.

Though chromium can exist in eleven valence states ranging from −4 to +6 [7], hexavalent chromium [Cr(VI)] and trivalent chromium [Cr(III)] show major ecological significance because of their stability in the natural environment. Hexavalent oxyanions (HCrO_4_^−^, CrO_4_^2−^, and  Cr_2_O_7_^2−^) and trivalent cations (Cr^3+^ and CrOH^2+^) are the prevalent species of chromium in industrial effluents. Its speciation is dependent on the pH. Hexavalent chromium is known to have 100-fold more toxicity than trivalent chromium because of its higher water solubility, mobility, and oxidizing power. It can act as carcinogen, mutagen, and teratogen in biological systems [8, 9].

Traditional processes for the removal of chromium from liquid effluents include methods such as ion exchange [10], electrochemical precipitation [11], solvent extraction [12], chemical precipitation [13], or membrane separation [14]. However, these processes are not ecofriendly and suffer from drawbacks such as high operating and maintenance costs, incomplete metal removal, high energy requirements, ineffectiveness at low concentrations of metal ions, and generation of toxic waste products requiring safe disposal [15]. Due to increase in legal constraints policies on discharge of effluents and environmental awareness, cost-effective alternative technologies as biosorption have been proposed [16].

Biosorption may be simply defined as the removal of substances from solution by biological material. Such substances can be organic or inorganic and in gaseous, soluble, or insoluble forms. Biosorption is gaining prominence as wastewater treatment process, producing high quality effluents which are low in metal ion concentrations [17]. The major advantages of biosorption over conventional treatment methods include lower price, high effectiveness, minimization of chemical and/or biological mud, restoration of biosorbent, and possibility of metal recovery. A large number of materials have been tested as biosorbents for hexavalent chromium removal including bacteria [18], fungi [19], algae [20], yeast [21], agricultural products [5], and other nonliving biomass as chitosan [22] or clays [23]. Natural materials that are available in large quantities or certain byproducts from the food and agricultural processing industries may have potential to be used as biosorbents, as they represent unused resources widely available [24].

*Opuntia* cladodes are a good low-cost candidate for utilization as biosorbent. They contain polysaccharide mucilage with varying proportions of galactose, arabinose, xylose, and rhamnose as well as galacturonic acid [25]. A similar composition has been reported for ectodermis of cactus pear fruits containing sugars such as galacturonic acid and rhamnose and features polysaccharides of pectin composition [26]. Their chemical composition reveals close resemblance with pectin, structural elements of primary cell walls, and intercellular regions of higher plants.

This paper presents the use of* Opuntia* biomass (cladodes and ectodermis from cactus pear fruits) as potential biosorbent for hexavalent chromium removal from aqueous solution. Parameters affecting the biosorption process are discussed. In addition, kinetic models and adsorption isotherms were tested in order to have a better understanding of the biosorption process.

## 2. Materials and Methods

### 2.1. Biosorbent Preparation

**Figure 1:**
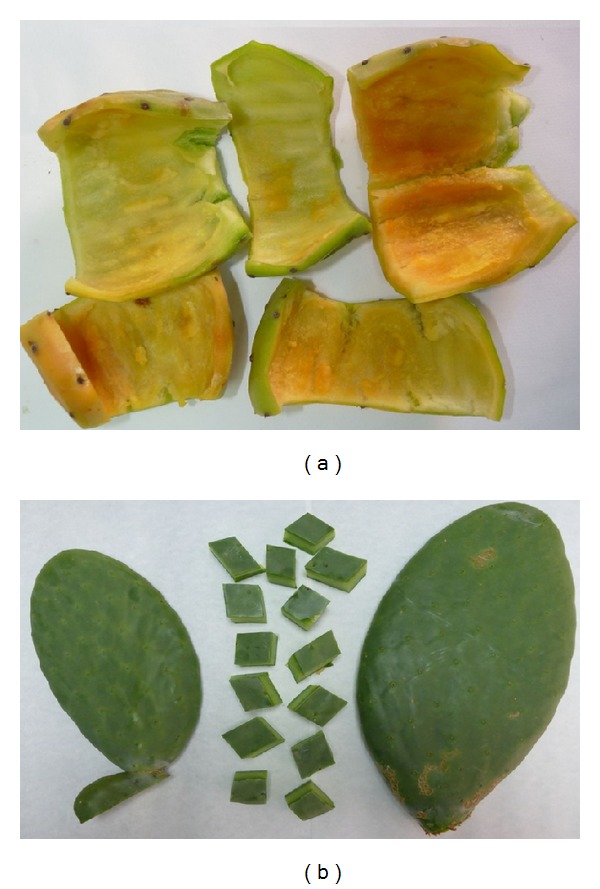
Ectodermis of cactus pear fruits (a) and* Opuntia* cladodes (b) used as biosorbents.

*Opuntia Cladodes*.* Opuntia* cladodes (Figure 1) were collected from a number of plants in Alhama (Murcia, Spain). They were washed repeatedly with water to remove dust and soluble impurities, cut in strips of 4 cm width, and dried at 60°C for 48 h. Dried material was grounded in a laboratory knife mill and sieved through a number 18 mesh (1.00 mm).

*Ectodermis of Cactus Pear Fruits*. Ectodermis (Figure 1) was obtained from mature* Opuntia ficus-indica* fruits harvested in Alhama (Murcia, Spain), washed with deionized water, and dried at 60°C for 48 hours. Then, dried material was grounded and sieved identically as* Opuntia* cladodes.

Protonated biomass was prepared by soaking 10 g of native biomass (cladodes and ectodermis) in 150 mL of 1 mol L^−1^ H_2_SO_4_ under magnetic stirring at slow agitation (30 rpm) for 24 h. After the acid treatment, the biosorbent was thoroughly washed with ultrapure water from a Milli-Q system (Millipore, Bedford, MA, USA), dried at room temperature for 48 h, and stored in a desiccator prior to use.

### 2.2. Preparation of Cr(VI) Solutions

The stock solution (1 g L^−1^) of Cr(VI) was prepared by dissolving 2.828 g of K_2_Cr_2_O_7_ in 1 L of deionized water. The working solutions were obtained by diluting the stock solution to appropriate volumes. The pH values were adjusted to desired values with 0.1 mol L^−1^ HCl or 0.1 mol L^−1^ NaOH solution by using Metrohm 654 pH meter with a combined pH electrode. Fresh diluted solutions were used for each experiment. All the chemicals used were of analytical grade.

### 2.3. Batch Biosorption Experiments

The influence of pH was studied at room temperature (20°C) by contact of the biosorbent (0.05 g) with 100 mL of Cr(VI) solution (10 mg L^−1^) at target pH values. The reaction mixture was agitated on a rotary shaker at 150 rpm for 24 h. After the contact time, solutions were filtered using 0.45 *μ*m pore size cellulose acetate membrane and the filtrate was analyzed by ICP in an Agilent 720/725 ICP-OES system (Agilent Technol., Santa Clara, CA, USA).

The mass balance equation was used for determining the sorption capacity *q* (mg Cr(VI) g^−1^) according to

(1) $$\begin{array}{c} q=\frac{\left(C_{0}-C_{e}\right)\times V}{m}, \end{array}$$

where *C*_0_ (mg L^−1^) is the initial Cr(VI) concentration, *C*_*e*_ (mg L^−1^) is the equilibrium concentration after the adsorption has taken place, *V* is the solution volume (L), and *m* is the dried* Opuntia* biomass (g) added. The final pH was systematically monitored at equilibrium.

### 2.4. Kinetic Studies

Uptake kinetics were determined at room temperature (20°C) mixing 0.5 L of Cr(VI) solution of concentration 10 mg L^−1^ at pH 2.0 with 0.5 g of biomass. The suspension was mixed on a rotary shaker at 150 rpm and samples were collected at different contact times, filtrated, and analyzed by ICP-OES for the determination of the kinetic profile. All the results obtained in the experiments were corrected from blanks performed under the same conditions but in the absence of biosorbent. All the results obtained represent the average from two replicate experiments.

### 2.5. Sorption Isotherm Models

Modeling of sorption isotherm data is important for predicting and comparing the sorption performance of the biosorbent. Therefore, the equilibrium data were fitted using different isotherm models, namely, Langmuir, Freundlich, and Temkin. Sorption isotherms were performed at pH 2.0 (optimum pH). A given amount of biomass (0.1 g) was dropped into 100 mL of Cr(VI) solution. The initial metal concentration was varied between 5 and 70 mg L^−1^. The suspension was maintained under agitation at room temperature (20°C) for 24 h using a rotary shaker at 150 rpm. Finally, the suspension was filtrated and the residual Cr concentration was analyzed by ICP-OES.

## 3. Results and Discussion

### 3.1. The Effect of pH on Cr(VI) Biosorption

**Figure 2:**
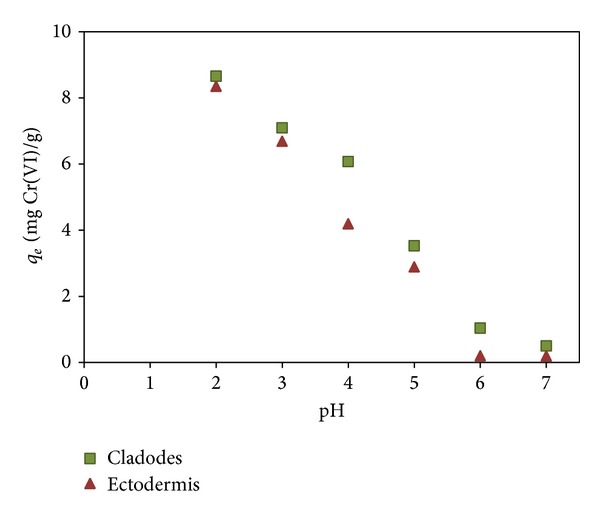
Effect of pH on Cr(VI) biosorption using* Opuntia* biomass (contact time: 24 h, sorbent dosage: 0.5 g L^−1^, and initial metal concentration: 10 mg L^−1^).

A pH study was done, in order to define the optimal pH of the chromium biosorption. Experiments over a range of pH values (2–7) with 10 mg/L of Cr(VI) concentration in solution in Figure 2 reveal that biosorption uptake of Cr(VI) with* Opuntia* biomass (cladodes and ectodermis) is clearly pH-dependent. As a result of the experiments, the highest biosorption was obtained at pH 2. The maximum adsorption of Cr(VI) in the lower pH range has been observed by many authors [20, 28]. The pH is an important parameter for biosorption processes since it affects the speciation of the metal (metal distribution, precipitation, and complexation), the stability of the biomass (potential degradation and leaching of some compounds and functional groups), and the chemical state of its reactive groups (protonation/deprotonation). Cr(VI) usually presents in different forms such as chromates (CrO_4_^2−^), dichromates (Cr_2_O_7_^2−^), and bichromates (HCrO_4_^−^) depending on pH and Cr(VI) concentration. Below pH 6, Cr(VI) is present in solution mainly as Cr_2_O_7_^2−^. As the pH decreases from 6 to 2, the concentration of Cr_2_O_7_^2−^ increases and, at the same time,* Opuntia* biomass becomes more positively charged, and so the adsorbed amount increases. So, at lower pH ranges, due to the high electrostatic force of attraction, the percentage of Cr(VI) removal is higher. Above pH 6, Cr(VI) exits in solution in the form of CrO_4_^2−^, increasing its concentration with the pH. At high pH ranges, negatively charged surface sites on the biosorbent do not favor the adsorption of ions due to electrostatic repulsions. Therefore, the possible mechanisms of metal ion sorption may be sorbent-sorbate interactions between the protonated adsorption sites of the biosorbent and the negatively charged sorbate species [4]. In the present work, the highest Cr(VI) uptake was obtained at pH 2 (Figure 2); at this pH, Cr(VI) anions can form complexes with protonated functional groups on the surface of the acidified* Opuntia* biomass such as –COOH –NH_2_ and –SO_3_H. From this result, pH 2 was defined as pH of work for the following experiences; this value allows combining favorable conditions for equilibrium pH (consistent with metal stability) and high adsorption yield.

### 3.2. The Effect of Biosorbent Dosage

**Figure 3:**
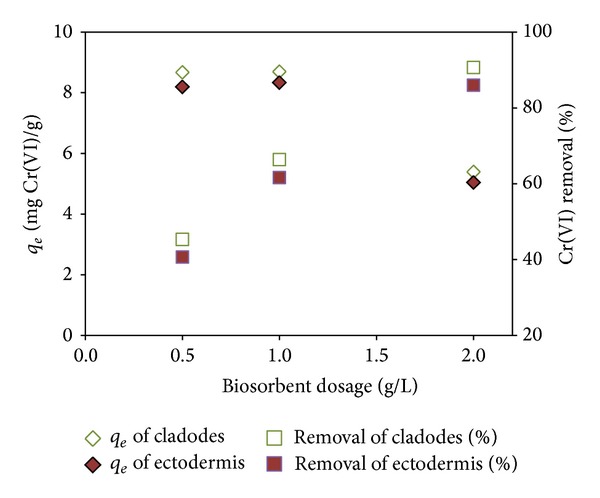
Effect of sorbent dosage on Cr(VI) biosorption with* Opuntia* biomass (contact time: 24 h and initial metal concentration: 10 mg L^−1^, pH 2).

The removal efficiency of metals is highly dependent on the quantity of the biosorbent. Several researches reported that the increase in the percentage removal with increase in the sorbent dosage is due to the greater availability of the exchangeable sites or surface area at higher concentration of the biosorbent [20, 36]. As revealed in Figure 3, the percentage removal increased with increase in biosorbent dose. However, the biosorption capacity was higher at low dose rates. The reason for this may be the availability of lesser binding sites and these were fully utilized. At the sorbent dosage of 2.0 g L^−1^, the uptake of the* Opuntia* cladodes was 5.1 mg Cr(VI) per gram of sorbent, clearly lower than at dosage of 0.5 or 1.0 g L^−1^ (8.7 mg Cr(VI) g^−1^).

Similar results were obtained when ectodermis of cactus fruits was used as biosorbent. The highest percentage removal (83%) was obtained at the sorbent dosage of 2.0 g L^−1^, while the biosorption capacity was higher at the sorbent dosage of 0.5 g L^−1^. The decrease of *q*_*e*_ with increase of biomass concentration might be due to the formation of aggregates between the biomass particles at high biomass concentration, reducing the effective adsorption area. Similar results were obtained for Pb(II) biosorption on* Opuntia* [38].

### 3.3. The Effect of the Initial Metal Concentration

**Figure 4:**
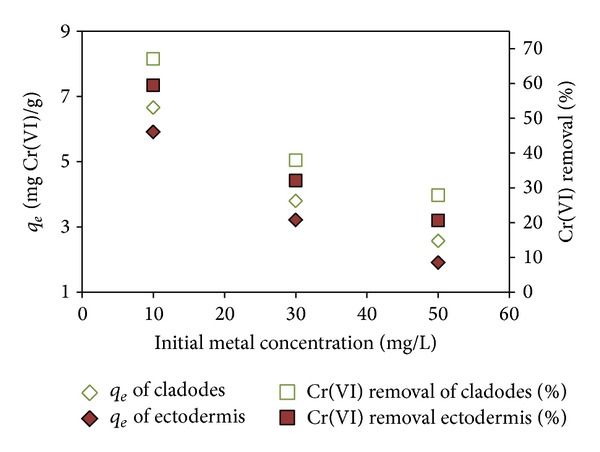
Effect of initial Cr(VI) concentration on Cr(VI) biosorption (contact time: 24 h and sorbent dosage: 1 g L^−1^, pH 2).

The efficiency of metallic biosorption for different initial Cr(VI) concentrations (from 10 mg L^−1^ up to 50 mg L^−1^) was investigated by carrying out biosorption experiments at the best experimental conditions: pH 2.0 and biomass concentration of 1.0 g L^−1^. The initial concentration generates an important driving force to overcome all mass transfer resistance of Cr(VI) between the aqueous and solid phases. Results (Figure 4) revealed that, increasing the initial Cr(VI) concentration, the uptake decreased both for* Opuntia* ectodermis and* Opuntia* cladodes and the highest *q*_*e*_ values were obtained with an initial Cr(VI) concentration of 10.0 mg L^−1^. Since biosorbent particles offer a finite number of surface binding sites, uptake showed saturation at higher metal ion concentrations.

### 3.4. Uptake Kinetic

**Figure 5:**
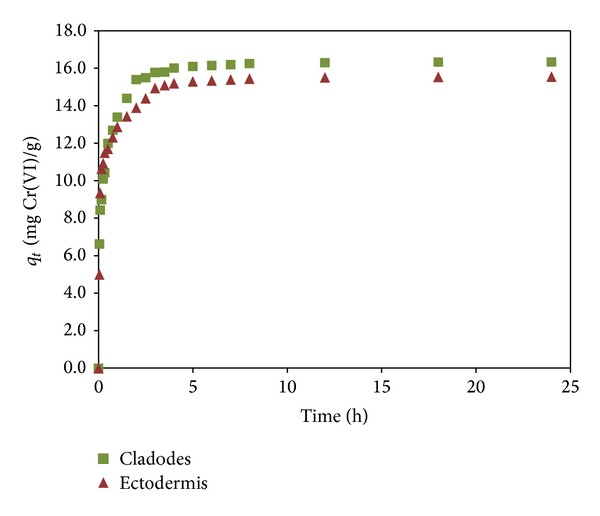
Kinetics of Cr(VI) biosorption using* Opuntia* biomass (sorbent dosage: 1.0 g L^−1^ and initial metal concentration: 10 mg L^−1^, pH 2).

From Figure 5, a two-stage kinetic behavior is evident for both sorbents: a rapid initial sorption over a 5 h, followed by a long period of much slower uptake. In general, more than 90% of the total metal ion sorption was achieved within 5 h. The magnitude of the experimental *q*_max⁡_ values obtained was 16.3 mg Cr(VI) g^−1^ for cladodes and 15.6 mg Cr(VI) g^−1^ for ectodermis. In order to analyze the sorption rates of Cr(VI) onto* Opuntia* biomass, three models were tested, the pseudo-first-order model [39], the pseudo-second-order model [40], and the intraparticle diffusion model [41].

The pseudo-first-order rate equation (PFORE) or the so-called Lagergren equation can be expressed as

(2) $$\begin{array}{c} \frac{{dq}_{t}}{dt}=k_{1}\cdot \left(q_{e}-q_{t}\right), \end{array}$$

where *q*_*e*_ and *q*_*t*_ (mg g^−1^) are the metal uptake at equilibrium and at time *t*, respectively, and *k*_1_ (h^−1^) is the pseudo-first-order constant of biosorption.

The rate law equation also can be considered a pseudo-second-order (PSORE) chemical biosorption process with respect to the sorbent sites, and it is expressed as

(3) $$\begin{array}{c} \frac{{dq}_{t}}{dt}=k_{2}\cdot {\left(q_{e}-q_{t}\right)}^{2}, \end{array}$$

where *q*_*e*_ and *q*_*t*_ (mg g^−1^) are the metal uptake at equilibrium and at time *t*, respectively, and *k*_2_ (g mg^−1^ h^−1^) is the pseudo-second-order constant of biosorption. The sorption rate *v*_0_ = *k*_2_ · *q*_*e*^2^_ (mg g^−1^ h^−1^) can be regarded as the initial sorption rate as *t* approaches 0. This model is based on the assumption that the rate-limiting step is chemisorption involving sharing or exchanging electrons between sorbent and sorbate. The existence of other processes, such as intraparticle diffusion, mass transfer, or ion interaction, is not taken into account. Though experimental data yielded a good fit to this simplified model, it should be noted that the model assumes that all sorption sites are homogeneous and does not consider the heterogeneous nature of the biomass.

When the intraparticle diffusion is the rate-limiting step, the uptake of the sorbate varies with the square root of time as

(4) $$\begin{array}{c} q_{t}=k_{d}\cdot t^{1/2}, \end{array}$$

where *k*_*d*_ is the internal diffusion coefficient (mg g^−1^ h^−1/2^) and *q*_*t*_ is the amount of metal adsorbed (mg g^−1^) at time *t* (h). The correlation coefficient values for this model were low, indicating that pore diffusion was not the controlling step.

The kinetic rate constants obtained from pseudo-first-order, pseudo-second-order, and intraparticle diffusion models are given in Table 1. Although both pseudo-first-order and pseudo-second-order kinetics present high correlation coefficients, the experimental *q*_*e*_ values obtained for cladodes and ectodermis are closer to those calculated for the second-order model. The physical structure and chemical components of the biosorbent determine the adsorptive behaviour, which can be attributed to various mechanisms. We can concluded that Cr(VI) biosorption onto* Opuntia* biomass seems to be more pseudo-second order (Figure 4), suggesting a predominant chemical reaction mechanism. Similar results were reported on Cr(VI) uptake by* Sargassum muticum* [20],* Ficus carica* [33], and* Tamarindus indica* [36].

### 3.5. Sorption Isotherms

Analysis of the isotherm data is important in order to develop an equation which accurately represents the results and which could be used for design purposes. The sorption data obtained from experiments provide information of maximum adsorption capacity of the biosorbent and effectiveness of sorbate-biosorbent system. The sorption capacity and other parameters were assessed using Langmuir, Freundlich, and Temkin models.

Table 2 summarizes the isotherm constants and correlation coefficients obtained. The Langmuir isotherm presupposes monolayer adsorption onto a surface containing a finite number of adsorption sites via uniform strategies of adsorption with no transmigration of the sorbate taking place along the plane of the surface. The linear form of the Langmuir isotherm model is given by the equation

(5) $$\begin{array}{c} \frac{1}{q_{e}}=\frac{1}{q_{\max}}+\frac{1}{K_{L}\cdot q_{\max}\cdot C_{e}}, \end{array}$$

where *q*_max⁡_ (mg g^−1^) and *K*_*L*_ (L mg^−1^) are the Langmuir constants related to adsorption capacity and rate of adsorption, respectively, *q*_*e*_ is metal ion concentration at equilibrium onto biosorbent (mg g^−1^), and *C*_*e*_ is metal ion concentration at equilibrium in solution (mg L^−1^). The applicability of Langmuir isotherm assumes a monolayer coverage and uniform activity distribution on the biosorbent surface. The *K*_*L*_ value determined is further used to calculate the dimensionless separation factor (*R*_*L*_) which is given as

(6) $$\begin{array}{c} R_{L}=\frac{1}{\left(1+K_{L}\cdot C_{0}^{\prime }\right)}, \end{array}$$

where *C*_0_′ is the highest initial concentration examined (mg L^−1^). The magnitude of *R*_*L*_ gives an idea about the nature of sorption equilibrium. If *R*_*L*_ < 1.0, a favourable sorption is considered. The *R*_*L*_ values of 0.14 (cladodes) and 0.16 (ectodermis) indicate that* Opuntia* biomass is a suitable biosorbent for the sorption of Cr(VI) from aqueous solution. The *q*_max⁡_ value is the maximum value of *q*_*e*_, which is important to assess the highest uptake capacity, and, as such, is useful in scale-up considerations. The magnitudes of *q*_max⁡_ were 18.5 and 16.4 mg g^−1^ for cladodes and ectodermis, respectively. These values are comparable to those reported previously on different heavy metals [38, 42, 43].

The Freundlich isotherm assumes a heterogeneous surface energy for which the energy term in the Langmuir equation varies as a function of surface coverage. The logarithmic form of the Freundlich isotherm is expressed as

(7) $$\begin{array}{c} \ln q_{e}=\ln K_{F}+\frac{1}{n}\ln C_{e}, \end{array}$$

where *K*_*F*_ (mg g^−1^) and *n* are Freundlich constants, with *n* giving an indication of the facility with which the adsorption process takes place. The values of *n* > 1 observed for both biosorbents (Table 2) indicated favourable and heterogeneous sorption. These results imply that monolayer biosorption, as well as heterogeneous surface conditions, may coexist under the applied experimental conditions. Hence, the overall sorption of Cr(VI) on* Opuntia* biomass is complex, involving more than one mechanism, such as ion exchange, electrostatical attraction, and surface complexation [20, 44].

Temkin isotherm takes into account sorbate-sorbent interactions and assumes that fall in the heat of sorption is linear rather than logarithmic, as implied in Freundlich equation. The Temkin relationship in linear form is given as

(8) $$\begin{array}{c} q_{e}=\frac{RT}{b}\ln\alpha +\frac{RT}{b}\ln C_{e}, \end{array}$$

where *T* is the absolute temperature (K), *R* is the universal gas constant (8.314 J mol^−1^ K^−1^), and *b* is the Temkin constant related to heat of adsorption (J mg^−1^). The Temkin constants *α* and *b* are calculated from the slope and intercept of *q*_*e*_ versus ln⁡⁡*C*_*e*_.

The biosorption isotherms obtained for Cr(VI) ion uptake by* Opuntia* biomass were found satisfactory to both the Langmuir and Freundlich predictions within the studied metal concentration range (5–70 mg L^−1^).

### 3.6. Comparison of* Opuntia* Biomass with Other Biosorbents

The biosorption capacity of Cr(VI) onto* Opuntia* biomass was compared with different low-cost biosorbents reported in the literature (Table 3). It is worthwhile mentioning that a critical direct comparison of sorbents is difficult due to dissimilar experimental conditions such as temperature, pH, and sorbent dosage. However, our results would confirm that both of the biosorbents studied (cladodes and ectodermis) possess reasonable adsorption capacity of hexavalent chromium in comparison with other low-cost biosorbents.

## 4. Conclusions

It is known that it is expensive and ineffective to remove Cr(VI) ions from aqueous solutions using conventional methods when the chromium concentration is low (1–100 mg L^−1^). A biosorption process with* Opuntia* biomass, an ecofriendly and low-cost sorbent, is a method that could replace conventional processes for remediating Cr(VI) pollution in aqueous systems. In the light of experimental results obtained and their evaluation, cladodes and ectodermis from cactus fruits, and abundantly available* Opuntia* biomass, could be considered a potential biosorbent for the removal of Cr(VI) from aqueous solutions. The behavior of both sorbents was quite similar. The percentage removal was found to depend on the quantity of biosorbent, time, and initial concentration of the sorbate. The process of uptake was strongly dependent on pH, with maximum biosorption capacity obtained at pH 2. Pseudo-second-order kinetics model was found to be the predominant. The equilibrium biosorption data fitted both the Langmuir and Freundlich isotherms with high correlation coefficients, suggesting that the process followed a monolayer biosorption.

## Figures and Tables

**Table 1:** Kinetic parameters for biosorption of Cr(VI) on *Opuntia* biomass.

Kinetic model	*Opuntia* biomass	
	Cladodes	Ectodermis
PFORE		
*q*_*e*_	17.446	19.893
*k*_1_	0.273	0.265
*R* ^2^	0,965	0.943
PSORE		
*q*_*e*_	16.207	15.015
*k*_2_	0.656	0.765
*v*_0_	108.696	172.414
*R* ^2^	0.970	0.957
Intraparticle diffusion		
*k*_*d*_	2.333	2.053
*R* ^2^	0.536	0.491

Sorbent dosage: 1.0 g L^−1^ and Cr(VI) concentration: 10 mg L^−1^, pH 2.0.

**Table 2:** Isotherm model constants for biosorption of Cr(VI) on *Opuntia* biomass.

*Opuntia* biomass	Langmuir			Freundlich			Temkin		
	*q*_max⁡_	*K*_*L*_	*R* ^2^	*n*	*K*_*F*_	*R* ^2^	*b*	*α*	*R* ^2^
Cladodes	18.518	0.087	0.922	2.759	1.642	0.954	4343.3	1.041	0.896
Ectodermis	16.434	0.074	0.966	2.417	1.428	0.996	4740.7	1.370	0.961

Temperature: 20°C, pH 2.

**Table 3:** Biosorption capacity of Cr(VI) on different low-cost biosorbents.

Biosorbent	*q*_max⁡_ (mg g^−1^)	Reference
Almond green hull	2.04	[27]
Rice straw	3.15	[28]
Almond shell	3.40	[29]
Groundnut shell	5.88	[29]
Coconut coir	6.30	[30]
Maize cob	13.80	[31]
Sawdust	15.82	[32]
*Ficus carica* fiber	19.68	[33]
Pine needles	21.50	[32]
Eucalyptus bark	45.00	[34]
Tea factory waste	54.65	[35]
Tamarind fruit shells	74.62	[36]
Walnut hull	98.13	[37]
Cladodes (*Opuntia* biomass)	18.51	This study
Ectodermis (*Opuntia* biomass)	16.43	This study
